# The Possible Role of Antibodies in Alopecia: A Narrative Review

**DOI:** 10.3390/antib15020031

**Published:** 2026-04-03

**Authors:** Julia Cieślawska, Mariola Pawlaczyk, Justyna Gornowicz-Porowska

**Affiliations:** Department and Division of Practical Cosmetology and Skin Diseases Prophylaxis, Poznan University of Medicinal Sciences, Rokietnicka 3, 60-806 Poznań, Poland; mariolapawlaczyk@ump.edu.pl (M.P.); justyna.gornowicz-porowska@ump.edu.pl (J.G.-P.)

**Keywords:** alopecia, immunopathogenesis, antibodies

## Abstract

Human hair performs a number of important physiological and esthetic functions. Hair loss and alopecia are complex disorders which affect people all over the world. Hair loss can be an early manifestation of various autoimmunological disorders. Despite a growing interest of researchers in the role of immune factors—especially autoantibodies—in the etiology of certain types of alopecia, their role in alopecia remains uncertain. Several potential autoantigens of follicular components, mainly derived from keratinocytes and melanocytes of the hair follicles, have been found to play a role in the development of alopecia areata. The list of autoantigens includes trichohyalin, keratin 16, fibroblast growth factor receptor 3, glycoprotein-100, melanoma-associated antigen recognized by T cells 1, dopachrome tautomerase/tyrosinase-related protein 2, tyrosinase, and tyrosine hydroxylase. This narrative review presents different aspects of immunopathogenesis of alopecia, from physiology (hair follicle immune privilege) to pathology (disruption of hair follicle immune privilege) and signaling pathways. Identification of key autoantigens could potentially pave the way for the development of new, effective, and more targeted immunotherapies for alopecia.

## 1. Introduction

Hair performs various physiological and esthetic functions: it protects the skin from different kinds of injury and environmental factors, it helps to maintain thermal homeostasis, and it plays a significant role in social communication [1,2,3]. The scalp contains approximately 100,000–150,000 hair follicles, while the entire human body has around 5 million hair follicles [4]. Mean hair growth has been estimated at approximately 0.37–0.44 mm/day, with a higher growth rate observed in males as compared to females [5,6].

A fully developed hair shaft originates from a hair follicle (HF). The hair is embedded in the skin by its root, while the portion protruding above the skin surface is known as the shaft, which is composed of dead, keratin-filled cells, and consists of the cuticle, cortex, and medulla [4]. The HF is a skin appendage which is formed during embryonic development. As far as structure is concerned, the HF is divided into three parts: the infundibulum, the isthmus, and the lower segment, which includes the hair bulb where the dermal papilla (DP) is located [7]. The DP has a dual function: it supplies nutrients through a network of blood vessels and regulates the hair growth cycle. It interacts with stem cells and participates in the processes of cell proliferation and differentiation within the follicle via the WNT signaling pathway. A fully developed, mature HF is a structure composed of multiple layers of epithelial cells and specialized papilla cells derived from the dermis. Skin stem cells, through local signaling mechanisms, activate HF epithelial stem cells, which determines the cyclic and asynchronous nature of hair growth phases [7,8,9]. The hair growth cycle consists of three phases: the growth phase—anagen, the transition phase—catagen, and the resting phase—telogen, after which the HF re-enters the growth phase [10] (Table 1).

Disturbances in the hair growth cycle or irregular transitions between its phases may result in alopecia, affecting individuals of both sexes at various stages of their life [12]. The aim of this narrative review is to critically evaluate the role of antibodies in the immunopathogenesis of alopecia with particular emphasis on alopecia areata.

## 2. Review Methodology

This narrative review is based on selective analysis of the literature retrieved from databases such as PubMed and Google Scholar. The selection focused on studies addressing the immunological mechanism involved in alopecia, particularly those investigating antibodies, B-cell responses and the immune privilege of the hair follicle. Priority was given to recent publications and studies providing mechanism insights.

## 3. Characteristics of Alopecia

Hair loss and alopecia constitute a complex disorder, affecting individuals worldwide. Hair density on the scalp depends on the following: genetic factors, developmental stage, environmental factors, and the region of the scalp. An individual sheds between 70 and 100 hairs daily, mainly during washing and brushing. Persistent hair loss which exceeds that range is suggestive of an underlying pathological condition and warrants a dermatological or trichological consultation [13].

Alopecia is an umbrella term which encompasses a broad spectrum of hair loss disorders that may affect the scalp or, in some cases, the entire body. The term alopecia comes from the Greek word for ‘fox’ and was used in ancient Greek literature to describe hairless patches found on the fur of mangy foxes [14]. Alopecia may be transient or permanent and affects individuals of all ages and sexes. Despite its heterogeneity, alopecia remains one of the most prevalent dermatological conditions globally. Its steadily increasing incidence continues to pose significant clinical and public health concern. The role of immune factors in the etiology of certain types of alopecia has been emphasized [15].

Two main types of alopecia have been distinguished: cicatricial alopecia (CA) and non-cicatricial alopecia (non-CA), the latter being potentially reversible. CA results from the permanent destruction of the hair follicles, often due to autoimmune processes, inflammatory conditions, or trauma, while in non-CA, the follicular openings remain preserved, allowing for potential hair regrowth [8,16]. CA is further classified into primary and secondary subtypes. Primary CA occurs in aplasia cutis congenita or nevi, and is characterized by direct, inflammatory damage to the hair follicles, which leads to their irreversible destruction. Histopathological examination reveals inflammatory infiltrates (lymphocytic, neutrophilic, or mixed), follicular destruction, and loss of follicular openings. Cutaneous lesions are often accompanied by certain subjective symptoms: burning, pruritus, and scalp tenderness. The most commonly reported diseases associated with CA include lichen planopilaris, frontal fibrosing alopecia, discoid lupus erythematosus, and folliculitis decalvans (Table 2) [17,18,19,20,21]. Secondary cicatricial alopecia develops as a consequence of chronic inflammation secondary to external factors, i.e., trauma, infection, or exposure to toxins. Follicular atrophy, formation of permanent scars, and irreversible hair loss due to externally induced damage are observed. Typical examples include traction alopecia and chemotherapy-induced alopecia [5,8,9,13].

The presence of intact hair follicles, which directly enables follicular regeneration once the causative factor is eliminated, is a defining feature of all forms of non-CA [22]. Typical histological features include follicular miniaturization and concomitant sebaceous pseudohyperplasia, while the mechanisms leading to hair loss remain heterogeneous (Table 3). In telogen effluvium, a predominance of follicles in the telogen phase is observed. Alopecia areata (AA) is characterized by a lymphocytic infiltrate, synchronization of multiple follicles within the same growth phase, and premature transition from anagen to telogen [23]. Diagnostic evaluation of non-CA relies primarily on the assessment of the follicle number and growth cycle, trichoscopic examination, and identification of inflammatory infiltrates and associated specific features [22,23,24,25,26].

### 3.1. Hair Follicle Immune Privilege and Its Collapse

In order to protect the immunogenic autoantigens of HFs (mainly associated with the process of melanogenesis) from immune recognition, the phenomenon of hair follicle immune privilege (HP IP) was developed. HP IP may promote peripheral immune tolerance because hair follicles in the anagen phase create an immunosuppressive microenvironment in the skin. The distal parts of the hair follicle are surrounded by immunosuppressants, including MSH, TGF-β1, IGF-1, and IL-10, additionally supported by the inhibitory action of the NK cells [42]. In addition, the hair follicle epithelium is enriched with T lymphocytes and Langerhans cells and is surrounded by mast cells and macrophages [39]. When a pathological factor has been activated, the expression of class I and II major MHC molecules is observed throughout the follicular epithelium, including the papilla. There is an infiltration of the CD4+ and CD8+ T cells. Based on the literature review, the authors concluded that disease induction is associated with the collapse of HF immune privilege in both humans and animal models. Moreover, it has been demonstrated that AA in an animal exhibits a histological pattern similar to that observed in a human, which supports the validity of comparing findings obtained from both models [42]. As a result of the action of the inflammatory cytokines, mainly substance P and IFN-γ, the immune privilege of the HF is broken [43]. The autoimmune basis is believed to be the cause of AA symptoms in genetically predisposed individuals [39]. As in the case of AA, an abnormal immune response is a factor causing cicatricial alopecia. However, unlike AA, functional HF stem cells (eHFSCs) are also destroyed. Stem cells are responsible for maintaining the hair cycle and are located in the bulge of the hair bulb next to the attachment of the arrector pili muscle. Complete destruction of the eHFSCs leads to irreversible hair loss [39]. Functionality and failure of HF IP have become recognized as key factors in the pathobiology of several inflammatory hair loss disorders, e.g., primary cicatricial alopecia, and alopecia areata [44]. Autoimmunity-related hair loss has been postulated to involve T cell reactivity against HF proteins. However, there is still scant evidence regarding the precise role of specific antigens [45,46,47].

### 3.2. Autoantibodies in Alopecia

Hair loss can be an early manifestation of various autoimmunological disorders, like thyroiditis, diabetes mellitus, autoimmune polyglandular syndrome type I (APS I), atopic dermatitis, vitiligo, psoriasis, and lupus erythematosus. This association may be considered a potent indicator of the role of autoimmunity in the pathogenesis of alopecia, especially alopecia areata [48,49,50,51]. Alopecia areata has been considered an autoimmune disease due to an aberrant T cell response against hair follicle self-antigens [52]. Recently, in an interesting transcriptomic study, Borcherding et al. observed clonal expansion of the CD4+ and CD8+ T cells with shared clonotypes in different transcriptional states in AA in mice and humans, suggesting an autoantigen-dependent autoimmune response of cells in AA [53].

**Figure 1:**
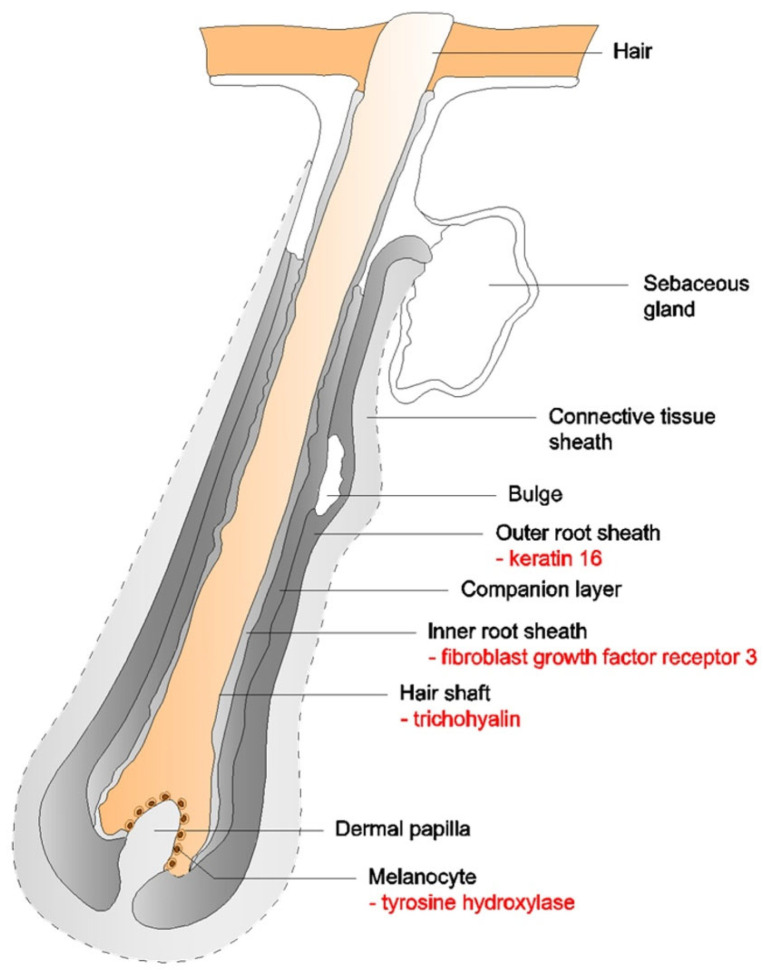
Possible locations of hair follicle autoantigens in alopecia areata. This figure presents an original schematic illustration.

Several potential autoantigens of follicular components, mainly derived from keratinocytes and melanocytes of the hair follicles, have been suggested to contribute to the development of alopecia areata. The majority of serum IgG antibodies detected in patients with AA target hair follicle antigens with a molecular weight of 40–60 kDa and ~220–250 kDa. The list of autoantigens includes trichohyalin, keratin 16, fibroblast growth factor receptor 3, glycoprotein-100 (gp100), melanoma-associated antigen recognized by T cells 1, dopachrome tautomerase/tyrosinase-related protein 2, tyrosinase, tyrosine hydroxylase [46,49,50,51,52,53,54,55,56]. Possible locations of hair follicle autoantigens in alopecia areata are shown in Figure 1 (own work based on [55,57,58,59]).

It is important to distinguish between the initial stage of AA and the antigens/epitopes that emerge during the antigenic drift and epitope spreading as AA progresses and/or in cases of long-term disease. Šutić Udović et al. [51] report that oxidative stress may activate NKG2D ligands, disrupt HP IP, and promote autoimmunity in AA patients. Jadeja et al. [50] examined posttranslational modifications of potential hair follicle autoantigens, as these modifications may alter protein antigenicity and contribute to the pathogenesis of alopecia areata. Therefore, the discovery of key HF-associated autoantigens which are actually responsible for triggering the initial immunologically cascade against HF in AA may help us better understand the onset and the course of the disease, as well as contribute to more effective characterization of the affected patients. Furthermore, the identification of key autoantigens could potentially pave the way for the development of new, effective, and more targeted immunotherapies.

Taken together, these findings highlight the central role of immune dysregulation and autoantigen-driven responses in the pathogenesis of AA, thereby providing a strong rationale for the development of targeted immunomodulatory therapies.

### 3.3. Targeting Immune Signaling in Alopecia

Immunopathogenic mechanisms and immunotherapeutic approaches have been explored in the management of various forms of alopecia; however, their application appears to be most extensively studied and best documented in AA.

Recently, preclinical and clinical data have shown the relatively high efficacy of Janus kinase (JAK) inhibitors in the treatment of AA [60]. JAK-STAT inhibitors can modulate the immune system by reducing inflammation and cytokine production. Immunopathogenesis of AA appears to be associated with increased expression of IFN-γ, which may contribute to the activation of the JAK–STAT signaling pathway. This process is thought to promote upregulation of MHC class I and II molecules in the hair follicle, potentially rendering it more susceptible to recognition and attack by NKG2D^+^ CD8^+^ T cells. In addition, pro-inflammatory cytokines such as IFN-γ, IL-2, and IL-15, acting through JAK–STAT signaling, are believed to play a role in the maintenance and amplification of inflammatory responses in AA, although their precise contribution remains to be fully elucidated [61].

JAK inhibitors, which belong to targeted therapies, can blockade the signaling pathway of AA by inhibiting JAK/STAT activation, leading to the reversal of AA. They can be used in topical and systemic therapy, and it is observed that, in the short term, about half of the patients achieve cosmetically satisfactory regrowth. JAK inhibitors used for the treatment of AA include: Baricitinib (inhibition of JAK1/2), Ritlecitinib (selectively inhibits JAK3 and TEC kinases), and Deuruxolitinib (inhibits JAK1/2) [62]. However, it is known that although the Th2 immune response is mainly driven by IL-4 and needs its downstream effector STAT6 for development, a number of recent studies provide evidence that pathways independent of IL-4/STAT6 signaling may induce Th2 differentiation in a complementary way. These include the Notch, mTORC2, and Wnt-signaling pathways [63]. Thus, while JAK inhibitors remain the current therapeutic standard, new antibody-based approaches are focusing on reversing immune privilege collapse and targeting specific cytokine pathways.

Another immunotherapy of AA involves diphenylcyclopropenone (DPCP), which is used to induce allergic contact dermatitis (through delay-type hypersensitivity), which allows hair to regrow through an unknown mechanism of action [64]. This may be due to antigenic competition that leads to local immunomodulation. Clinically, it enables hair regrowth by inducing an inflammatory response to contact sensitizing agents at the applied area. The second possible mechanism of action involves cytokine alteration, which increases in T-regulatory lymphocytes, causing a decline of the follicular immune reaction.

### 3.4. The Role of Antinuclear Antibodies in Alopecia

The literature offers numerous reports about the association between the antinuclear antibody (ANA) and alopecia [65,66,67]. It has been postulated that ANA positivity is linked to the pathogenesis of central centrifugal cicatricial alopecia [66]. Moreover, Manav et al. [62] hypothesize that the ANA may be the link between COVID-19 and subsequent alopecia. They detected ANA positivity in 40% of the patients exhibiting hair loss after COVID-19 infection; in patients with COVID-19-related hair loss, diffuse hair loss and ANA positivity may be related to the high antibody levels triggered by COVID-19. Therefore, hair loss after COVID-19 may be triggered by the release of the autoantibodies in response to the infection [68,69,70,71]. Interestingly, it seems that cytoplasmic ANA patterns may be essential markers for elucidating the pathogenesis of hair loss after COVID-19. Choi et al. [60] found that ANA positivity was present in 30.4% of the 105 patients with pattern hair loss (PHL), while ANA positivity did not correlate with the severity of PHL. Also, the preliminary findings of these authors suggest that AA patients show similar results, and that a positive ANA test was found for 35.3% of the 17 AA patients. Choi suggest that the development of the ANA might result from skin immune homeostasis in PHL. Consequently, the significance of ANA positivity should be taken into account, and the analysis of ANA titer (both quantitative and specific pattern) should be performed in alopecia patients in the future. Kim and Jang emphasize the importance of regular antibody follow-ups in ANA-positive patients with diffuse hair loss, as it could be essential for the early detection of systemic lupus erythematosus. In their opinion, monitoring ANA, anti-dsDNA, and lupus-specific antibodies allows for a timely intervention, improving patient outcomes [72].

### 3.5. Other Circulating Antibodies in Alopecia Areata

In a retrospective epidemiologic study on the role of the thyroid autoantibodies in AA [73], Kasumagić–Halilović compared the frequency of thyroid autoantibodies—thyroglobulin antibody (TgAb), and thyroid peroxidase antibody (TPAb)—in 70 AA patients and 30 healthy volunteers. Thyroid functional abnormalities were found in 8 (11.4%) AA patients. The number of thyroid autoantibodies was significantly higher in AA patients as compared to healthy controls. Positive autoimmune antibodies were associated with AA in 18 (25.7%) patients, with no significant relationship between disease severity and the presence of these antibodies. The study supports the previous reports on the association between AA and thyroid autoimmunity [47,74,75,76].

In a case–control study, Mokhtari et al. evaluated the frequency distribution of celiac autoantibodies (anti-gliadin IgA, anti-gliadin IgG, anti-tissue transglutaminase IgA) in patients with AA as compared to controls. They concluded that biological tests used to search for celiac antibodies do not show evidence which would confirm gluten intolerance in AA patients [72,77].

## 4. Conclusions

Despite the growing interest in the role of antibodies in alopecia areata, their exact contribution to disease pathogenesis remains incompletely understood. Antibody-associated immune mechanisms have been reported in various forms of alopecia; however, further studies are required to better define their significance. Future research should focus on well-designed human studies, functional validation of candidate autoantigens and clarification of whether antibody responses are photogenic or secondary. The identification of specific autoantigens and their corresponding autoantibodies may contribute to a deeper understanding of the pathogenesis of different forms of hair loss and may open new avenues for the development of more targeted immunotherapeutic strategies.

## Figures and Tables

**Table 1:** Comparison of hair growth phases [4,11].

Characteristics	Anagen	Catagen	Telogen
duration	3–8 years; 20–30 cycles per one hair follicle	2–4 weeks/transition phase	2–4 months
key processes	proliferation of matrix cells; pigmentation of the hair root; synthesis of keratin and trichohyalin; immune response	inhibition of protein synthesis; apoptosis of the hair matrix; loss of melanocyte activity; formation of telogen hair	distal displacement of the hair shaft; formation of a new hair germ; exogen—shedding of the hair shaft
regulatory factors	vascular endothelial growth factor (VEGF); insulin-like growth factor 1 (IGF-1); hepatocyte growth factor (HGF); epidermal growth factor (EGF); keratinocyte growth factor (KGF); tumor necrosis factor-α (TNF-α); fibroblast growth factor (FGF)	fibroblast growth factor-5 (FGF-5); interleukin-β; neurotrophins (NT-3, NT-4)	pro-inflammatory cytokines (IL-1α, IL-1β); fibroblast growth factor-5 (FGF-5); TNF-α and transforming growth factor-β (TGF-β)

**Table 2:** Comparative characterization of cicatricial alopecia types.

Type	Etiology/Pathogenesis	Clinical Presentation
Lichen planopilaris [17,18]	autoimmunologic factors (IgG anti-keratin 17), interfollicular inflammation → fibrosis	parietal plaques with silvery-white tubular scaling; milky-red areas; absence of follicular openings
Frontal fibrosing alopecia [19,20,21]	hormonal (post-menopause), autoimmunologic factors; frontal fibrosing alopecia	recession of the frontal hairline; hair thinning and eyebrow loss; isolated single hairs (“lonely hair sign”); epidermal hyperplasia; ivory-colored skin
Discoid lupus erythematosus [20]	autoimmunologic factors (ANA, anti-dsDNA); environmental factors (UV); genetic factors;	erythematous patches with plugged follicular openings; dilation of blood vessels
Folliculitis decalvans [17]	bacteria (*S. aureus*, *P. acnes*)	epidermal hyperplasia; perifollicular scaling forming a collar-like pattern; pustules with yellow discharge; white areas devoid of hair follicles

The arrow (→) indicates the direction of the process, specifically the progression from interfollicular inflammation to fibrosis.

**Table 3:** Comparative characterization of non-cicatricial alopecia types.

Type	Etiology/Pathogenesis	Clinical Presentation	Major Diagnostic Findings
Alopecia Androgenetica [27,28,29,30,31,32,33]	androgenetic factors (5α-reductase type 2 induces the formation of dihydrotestosterone); more common in men	miniaturization of hair follicles; recession of the hairline (in men: frontotemporal and vertex regions; in women: central scalp thinning); presence of dystrophic hairs	trichoscopy; Hamilton–Norwood scale; visible hair heterogeneity: the frontal region is characterized by the lowest mean hair shaft diameter, an increased proportion of vellus-type hairs (long, blunt-ended), a significant increase in the number of single-hair follicular units, and a higher number of “yellow dots”; severity of perifollicular pigmentation shows a clear dependence on location, with predominance in the frontal area
Telogen effluvium [28,34,35]	shortening of the anagen phase; etiology: infections, hormonal imbalance, stress, medications, inflammatory conditions of the hair-bearing scalp	acute onset with >30% hair shedding; dryness, lightening, loss of luster; symptoms of trichodynia (burning sensation, pain, pruritus in the absence of visible skin lesions)	hair pull test (pull test); trichoscopy reveals the thickest hairs in the frontal region and the thinnest in the occipital area; vellus hairs are characterized by short length and pointed tips; the highest proportion of single-hair follicular units is observed in the temporal regions
Anagen effluvium [27,36,37,38]	chemotherapy, radiation therapy, exposure to heavy metals; poor diet	loose anagen hair syndrome;	trichoscopy reveals visible thinning and the presence of isolated follicular units; short hairs measuring 3–6 cm
Alopecia areata [39,40]	autoimmunologic factors, genetic factors, infections	focal hair loss (oval or round patches); confluence of primary lesions; complete hair loss; exclamation mark hairs and necrotic hairs; ocular involvement and nail plate changes	trichoscopy reveals the presence of empty follicular openings, enabling differentiation from cicatricial alopecia
Trichotillomania [5,41]	psychogenic (compulsive pulling)	broken hairs, split hair shafts; tulip hairs	trichoscopy

## Data Availability

No new data were created or analyzed in this study. Data sharing is not applicable to this article.

## Abbreviations

The following abbreviations are used in this manuscript:

HF	Hair follicle
DP	Dermal papilla
CA	Cicatricial alopecia
non-CA	Non-cicatricial alopecia
AA	Alopecia areata
HP IP	Hair follicle immune privilege
JAK	Janus kinase
ANA	Antinuclear antibody
PHL	Pattern hair loss
